# Querying knowledge graphs in natural language

**DOI:** 10.1186/s40537-020-00383-w

**Published:** 2021-01-06

**Authors:** Shiqi Liang, Kurt Stockinger, Tarcisio Mendes de Farias, Maria Anisimova, Manuel Gil

**Affiliations:** 1 ETH Swiss Federal Institute of Technology, Rämistrasse 101, 8092 Zurich, Switzerland; 2 Zurich University of Applied Sciences, Obere Kirchgasse 2, 8400 Winterthur, Switzerland; 3 SIB Swiss Institute of Bioinformatics, Quartier Sorge-Bâtiment Amphipôle, 1015 Lausanne, Switzerland; 4 Department of Ecology and Evolution, University of Lausanne, Quartier Sorge-Bâtiment Biophore, 1015 Lausanne, Switzerland

**Keywords:** Natural language processing, Query processing, Knowledge graphs, SPARQL

## Abstract

Knowledge graphs are a powerful concept for querying large amounts of data. These knowledge graphs are typically enormous and are often not easily accessible to end-users because they require specialized knowledge in query languages such as SPARQL. Moreover, end-users need a deep understanding of the structure of the underlying data models often based on the Resource Description Framework (RDF). This drawback has led to the development of Question-Answering (QA) systems that enable end-users to express their information needs in natural language. While existing systems simplify user access, there is still room for improvement in the accuracy of these systems. In this paper we propose a new QA system for translating natural language questions into SPARQL queries. The key idea is to break up the translation process into 5 smaller, more manageable sub-tasks and use ensemble machine learning methods as well as Tree-LSTM-based neural network models to automatically learn and translate a natural language question into a SPARQL query. The performance of our proposed QA system is empirically evaluated using the two renowned benchmarks-the 7th Question Answering over Linked Data Challenge (QALD-7) and the Large-Scale Complex Question Answering Dataset (LC-QuAD). Experimental results show that our QA system outperforms the state-of-art systems by 15% on the QALD-7 dataset and by 48% on the LC-QuAD dataset, respectively. In addition, we make our source code available.

## Introduction

Over the past decade knowledge graphs have been increasingly adopted to structure and describe data in various fields like education, biology [1] or social media [2]. These knowledge graphs are often composed of millions or billions of nodes and edges, and are published in the Resource Description Framework (RDF). However, querying such knowledge graphs requires specialized knowledge in query languages such as SPARQL as well as deep understanding of the underlying structure of these graphs. Hence, a wide range of end-users without deep knowledge of these technical concepts is excluded from querying these knowledge graphs effectively.

This drawback has triggered the design of natural language interfaces to knowledge graphs to enable non-tech savvy users to query ever more complex data [3–5].

With the development of the Semantic Web, a large amount of new structured data has become available in the form of knowledge graphs on the web. Hence, natural language interfaces and in particular Question-Answering (QA) systems over knowledge graphs have gained importance [6].

Even though these QA systems significantly improve the usability of knowledge graphs for non-technical users, they are far from perfect. Translating from natural language to SPARQL is a hard problem due to the ambiguity of the natural language. For instance, the word “Zurich” could refer to the city of Zurich, the canton of Zurich or the company “Zurich Financial Services”. To provide the correct result, a QA system needs to understand the users’ intention. Moreover, knowledge graphs are typically very complex and thus exhaustively enumerating all possible answer combinations is often prohibitive.

To extract answers from a given knowledge graph, QA systems usually translate natural language questions into a formal representation of a query by using techniques from natural language processing, databases, information retrieval, machine learning and the Semantic Web [7]. However, the accuracy of these systems still needs to be improved and a significant amount of work is required to make these systems practical in the real-world [8].

In order to tackle this hard challenge of translating from natural language to SPARQL, one approach is to break down the problem into smaller, more manageable sub-problems. In particular, we can conceptualize the problem as a linear, modular pipeline with components like Named Entity Recognition (NER), Relation Extraction (RE) and Query Generation (QG). Consider, for instance, the query “How many people work in Zurich?”. The NER-component recognizes “Zurich” as an entity which could have the three meanings as mentioned above. The RE-component recognizes the relation “works”. Finally, the QG-component needs to generate a SPARQL query by taking into account the structure of the knowledge graphs. However, most implementations of QA systems over knowledge graphs are not subdivided into such independent components [9].

Recently, Frankenstein [10] was introduced introduced as a truly modular QA systems. Frankenstein decomposes the whole task into three sub-tasks, i.e. (1) Named Entity Recognition and Disambiguation, (2) Relation Extraction, and (3) Query Building. It then dynamically selects the best performing QA component on each sub-task from a collection of 29 reusable QA components. Afterwards, the QA pipeline is generated based on the selected components. The advantage of Frankenstein is that the components in the whole pipeline are reusable and exchangeable and therefore this modular approach enables different research efforts to tackle parts of the overall challenge.

**Fig. 1:**
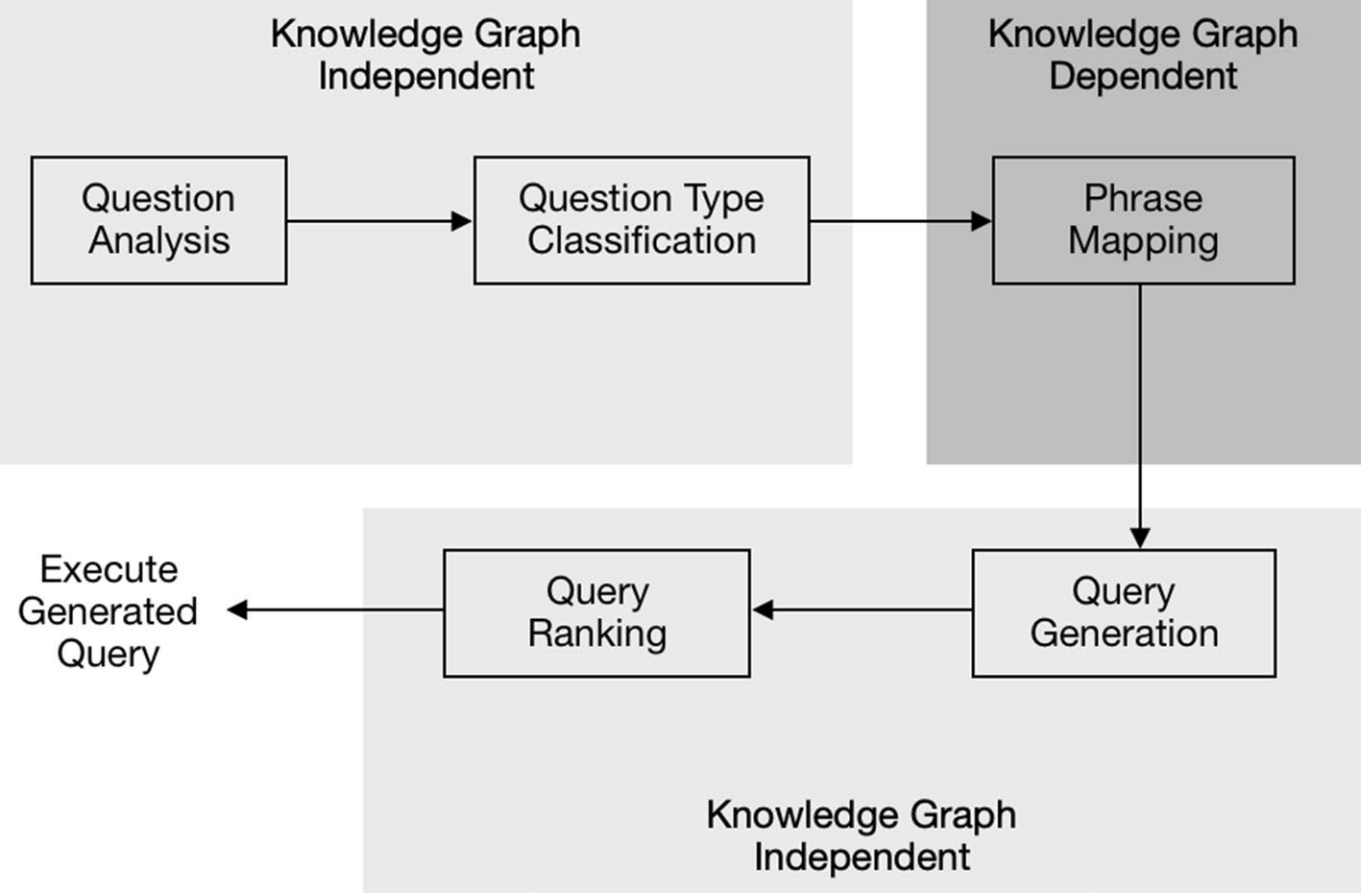
The architecture of the proposed QA system includes five components for five different tasks

In this paper we provide a novel modular implementation of a QA system. We build on the modular design of the Frankenstein framework [10] and the SPARQL Query Generator (SQG) [9]. At the most basic level, our system is structured into two parts: One part is *knowledge graph-dependent* and while the other part is *knowledge graph-independent* (see Fig. 1). The basic idea is to break up the task of translation from natural language to SPARQL in the following components: (1) Question analysis, i.e. syntactic parsing. (2) Question type classification, i.e. is it a yes/no questions or a count question? (3) Phrase mapping, i.e. mapping of entities and relationships in the natural language to the corresponding entities and relationships in the knowledge graph. (4) Query generation, i.e. construct a SPARQL query based on the entities and relationships identified in the knowledge graph. (5) Query ranking, i.e. rank the most relevant query as the highest. Details are discussed in "Methods" section.

To sum up, we introduce a *novel modular implementation of a QA system* based on the Frankenstein framework [10] and the SPARQL Query Generator (SQG) [9]. Through a careful design, including the choice of components, our system outperforms the state-of-the art, while requiring minimal training data. More specifically, we make the following main contributions:

- We subdivide our QA system into knowledge graph dependent and into knowledge graph independent modules. Like this, our QA system can be easily applied to newly unseen data domains. In particular, the independent modules (question type classification model and the query generation model) do not require any domain-specific knowledge.
- Large and and representative training data sets for RDF graphs are hard to devise [11]. In our system only the modules for question type classification and query ranking require training. As pointed out above, both modules are knowledge graph independent. Consequently, they can be trained on general purpose datasets. A training set of a few hundreds queries has been shown to be sufficient in our experiments (see "Results" section).
- In contrast to previous systems we use an *ensemble method for phrase mapping*. Moreover, question type classification is performed by a Random Forest Classifier, which outperforms previous methods.
- We extended the query generation algorithm in [9] to *include more complex queries*. Our system includes query ranking with Tree-structured Long Short-Term Memory (Tree-LSTM) [12] to sort candidate queries according to the similarity between the syntactic and semantic structure of the input question.
- We show that *our QA system outperforms the state-of-art systems* by 15% on the QALD-7 dataset and by 48% on the LC-QuAD dataset, respectively.
- We make our source code available (see "Results" section).

The paper is organized as follows. Related work" section gives an overview on the related work of QA systems on knowledge graphs. "Methods" section shows the architecture of our proposed system. "Results" section provides a detailed experimental evaluation including a comparison against state-of-the-art systems. Finally, "Discussion" section  concludes the paper and gives directions for future research.

## Related work

Building natural language interfaces to databases has been a long-standing research challenge for a few decades [13–15]. Early systems used rule-based, pattern-based or grammar-based approaches to translate from natural language to SQL [5, 7]. The introduction of the *Spider* leaderboard in 2018 has triggered a significant interest of several research groups to tackle the problem with machine learning approaches, in particular with advanced neural networks [16–19]. However, most of these systems provide solutions for translating from natural language to SQL rather than to SPARQL – which is the standardized query language for RDF graph databases.

Since our paper proposes a solution for querying knowledge graphs, we will now review the major work on QA systems over knowledge graphs such as [10, 20–22]. In particular, we focus our discussions on systems that are most relevant for understanding the contributions of our proposed QA system. Further comprehensive surveys on natural language interfaces to databases, including graph databases, were recently reviewed in [5, 23].

ganswer2 [20] answers natural language questions through a graph data-driven solution composed of offline and online phases. In the offline phase, the semantic equivalence between relation phrases and predicates is obtained through a graph mining algorithm. Afterwards a paraphrase dictionary is built to record the obtained semantic equivalence. The online phase contains question understanding stage and query evaluation stage. In the question understanding stage, a semantic query graph is built to represent the user’s intention by extracting semantic relations from the dependency tree of the natural language question based on the previously built paraphrase dictionary. Afterwards, a subgraph of the knowledge graph, which matches the semantic query graph through subgraph isomorphism, is selected. The final answer is returned based on the selected subgraph in the query evaluation stage. In contrast to ganswer2, our proposed system is component based. Our framework can be decomposed into independent components and therefore the overall accuracy can be improved by enhancing each component individually. As a result, our proposed system is much more flexible in terms of adapting to new techniques for question understanding and query evaluation.

WDAqua [21] is a QA component which can answer questions over DBpedia and Wikidata through both full natural language queries and keyword queries. In addition, WDAqua supports four different languages over Wikidata, namely English, French, German and Italian. WDAqua uses a rule-based combinatorial approach which constructs SPARQL queries based on the semantics encoded in the underlying knowledge base. As a result, WDAqua does not use a machine learning algorithm to translate natural language questions into SPARQL queries. Hence, WDAqua does not suffer from over-fitting problems. However, due to the limitations of human-defined transformation rules, the coverage and diversity of the generated SPARQL queries are limited. For instance, the generated SPARQL queries contain at most two triple patterns. Moreover, the modifiers in the generated queries are limited to the ‘COUNT’ operator. Adding a new operator in the generated queries would require significant work in designing the transformation rules. Instead, for machine learning-based systems, just collecting new question-answer pairs would be enough.

WDAqua-core1 [22] constructs queries in four consecutive steps: question expansion, query construction, query ranking and answer decision. In the first step, all possible entities, properties and classes in the question are identified through lexicalization. Then, a set of queries is constructed based on the combinations of the previously identified entities, properties and classes in four manually defined patterns. In the third step, the candidate queries are ranked based on five features including the number of variables and triples in the query, the number of the words in the question which are covered by the query, the sum of the relevance of the resources and the edit distance between the resource and the word. In the last step, logistic regression is used to determine whether the user’s intention is reflected in the whole candidate list and whether the answer is correct or not. There are mainly two differences between our proposed system and WDAqua-core1. Firstly, we use an ensemble method of state-of-the-art entity detection methods instead of using lexicalization. Therefore, the coverage of identified intentions is improved enormously. In addition, we use a Tree-LSTM to compute the similarity between NL questions and SPARQL queries as the ranking score instead of the five simple features selected by the authors of [22]. Hence, the final selected query is more likely to express the true intention of the question and extract the right answer.

Frankenstein [10] decomposes the problem into several QA component tasks and builds the whole QA pipeline by integrating 29 state-of-the-art QA components. Frankenstein first extracts features such as question length, answer, type, special words and part-of-speech (POS) tags from the input questions. Afterwards, a QA optimization algorithm is implemented in two steps to automatically build the final QA pipeline by selecting the best performing QA components from the 29 reusable QA components based on the questions. In the first step, the performance of each component is predicted based on the question features and then the best performing QA components are selected based on the predicted performance. In the second step, the QA pipeline is dynamically generated based on the selected components and answers are returned by executing the generated QA pipeline. Compared to Frankenstein, our proposed system uses an ensemble method instead of only selecting the best performing QA component. What is more, we use an improved version of the query construction component [9] other than selecting between the currently published QA components. ExSQG extends the former SQG to support more query types [24]. For instance, ExSQG supports ordinal questions such as superlatives, however it still does not consider a constraint that can be expressed within a filter clause.

## Methods

Here we describe the details of our proposed QA system. In particular, our system translates natural language questions to SPARQL queries in five steps (see Fig. 1 in "Introduction" section). At each step, a relevant task is solved independently by one individual software component. First, the input question is processed by the question analysis component, based solely on syntactic features. Afterwards, the type of the question is identified and phrases in the question are mapped to corresponding resources and properties in the underlying RDF knowledge graph. A number of SPARQL queries are generated based on the mapped resources and properties. A ranking model based on Tree-structured Long Short-Term Memory (Tree-LSTM) [12] is applied to sort the candidate queries according to the similarity between their syntactic and semantic structure relative to the input question. Finally, answers are returned to the user by executing the generated query against the underlying knowledge graph.

In the proposed architecture, only the Phrase Mapping is dependent on the specific underlying knowledge graph because it requires the concrete resources, properties and classes. All other components are independent of the underlying knowledge graph and therefore can be applied to another knowledge domain without being modified.

### Question analysis

The first component of our QA system analyzes natural language questions based solely on *syntactic features*. In particular, our system uses syntactic features to tokenize the question, to determine the proper part of speech tags of theses tokens, to recognize the named entities, to identify the relations between the tokens and, finally, to determine the dependency label of each question component [2].

Moreover, the questions are lemmatized and a dependency parse tree is generated. The resulting lemma representation and the dependency parse tree are used later for question classification and query ranking.

The goal of *lemmatization* is to reduce the inflectional forms of a word to a common base form. For instance, a question “Who is the mayor of the capital of French Polynesia?” can be converted to the lemma representation as “Who be the mayor of the capital of French Polynesia?”.

**Fig. 2:**
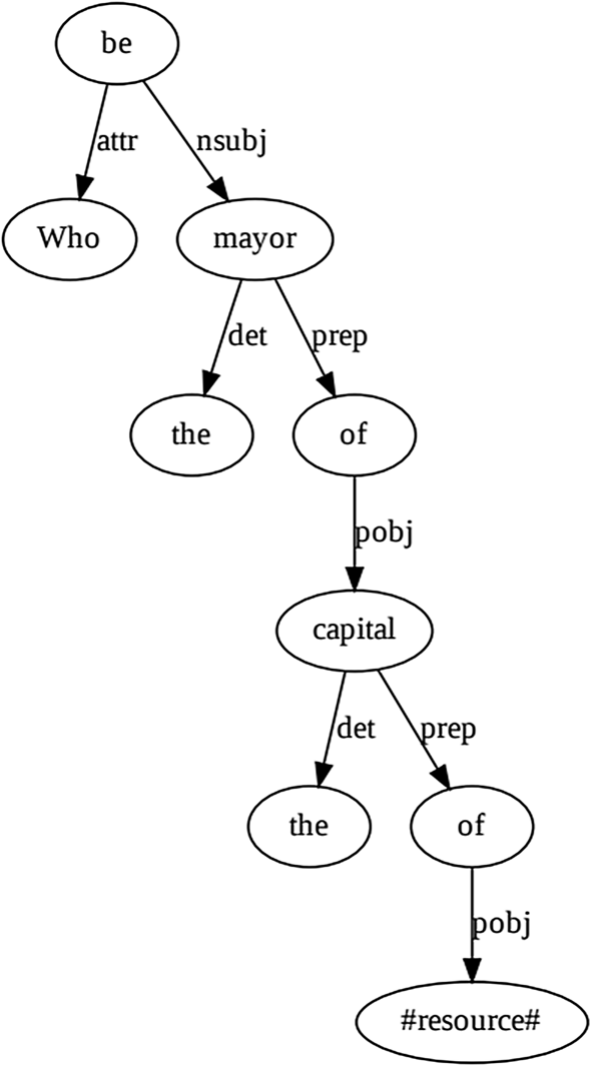
Lemma expressions and dependency parse tree annotated with dependency labels for the question: “Who is the mayor of the capital of French Polynesia?”

*Dependency parsing* is the process of analyzing the syntactic structure of a sentence to establish semantic relationships between its components. The dependency parser generates a dependency parse tree [25] that contains typed labels denoting the grammatical relationships for each word in the sentence (see Fig. 2 for an example).

### Question type classification

In order to process various kinds of questions such as ‘Yes/No’ questions or ‘Count’ questions, the proposed QA system first identifies the type of question and then constructs the WHERE clause in the SPARQL query. Our system currently distinguishes between three question types.

The first is the *‘List’ question* type, to which belong most common questions, according to our analysis of the available datasets (see "Results" section for details). ‘List’ questions usually start with a WH-word or a verb such as “list” or “show”. One example question could be ‘Who is the wife of Obama?’. The expected answer to the ‘List’ questions is a list of resources in the underlying knowledge graph.

The second type is the *‘Count’ question* type, where the keyword ‘COUNT’ exists in the corresponding SPARQL query. These kind of questions usually start with a particular word such as “how”. One example question could be ‘How many companies were founded in the same year as Google?’. The expected answer to a ‘Count’ question is a number.

Note that sometimes the expected answer to a ‘Count’ question could be directly extracted as the value of the property in the underlying knowledge graph instead of being calculated by the ‘COUNT’ SPARQL set function. For example, the answer of the question ‘How many people live in the capital of Australia?’ is already stored as the value of http://dbpedia.org/ontology/populationTotal. As a result, this question is treated as of the type ‘List’ instead of ‘Count’.

Finally, the *‘Boolean’ question* type must contain the keyword “ASK” in the corresponding SPARQL query. For example: ‘Is there a video game called Battle Chess?’. The expected answer is of a Boolean value - either True or False.

We use a machine learning method instead of heuristic rules to classify question types because it is hard to correctly capture all the various question formulations. For example, consider the question ‘How many people live in Zurich?’, which starts with ‘How many’ and belongs to question type ’LIST’ rather than ’COUNT’ (as in the example above). Similar questions include ’How high is Mount Everest’ which also belongs to question type ’LIST’. In order to capture those special questions, many specific cases must be considered while hand-crafting heuristic rules. Instead, using a machine learning algorithm for question type classification saves the tedious manual work and can automatically capture such questions as long as the training data is large and sufficiently diverse.

To automatically derive the question type, we first convert each word of the original question into its lemma representation. Then we use term frequency-inverse document frequency (TF-IDF) to convert the resulting questions into a numeric feature vector [26]. Afterwards, we train a Random Forest model [27] on these numeric feature vectors to classify questions into ‘List’, ‘Count’ and ‘Boolean’ questions. Our experimental results demonstrate that this simple model is good enough for this classification task (see Related work" section). Consequently, a SPARQL query will be constructed based on the derived question type. For instance, ‘ASK WHERE’ is used in the SPARQL query of a ‘Boolean’ question - rather than ‘SELECT * WHERE’.

### Phrase mapping

After the question types are identified, our QA system builds the final queries using the information related to the underlying knowledge graph. There are mainly three types of information when considering the RDF schema 1.1 to support the writing of SPARQL queries: resources, properties and classes [28].

- *Resources* are concrete or abstract entities denoted with any Internationalized Resource Identifier (IRI)^1^ or literal^2^. For instance, the IRI http://dbpedia.org/resource/Zurich. represents the city ‘Zurich’ or the string literal “CH-ZH” that denotes the Zurich region code in DBpedia.
- *Properties* are special resources used to describe attributes or relationships of other resources. For instance, the property http://dbpedia.org/ontology/postalCode. represents the postal code of a place.
- *Classes* are also resources. They are identified by IRIs and may be described with properties. For example, http://dbpedia.org/resource/Zurich. belongs to the class http://dbpedia.org/resource/City.

For phrase mapping our QA system uses an ensemble method, combining the results from several widely used phrase mapping systems. The ensemble method allows to overcome the weaknesses of each system while at the same time maximizing their strengths, so as to produce the best possible results.

For instance, in order to identify *Resources* in a natural language question, we use DBpedia Spotlight [29], TagMe [30], EARL [31] and Falcon [32]. In order to identify *Properties* we use EARL [31], Falcon [32] and RNLIWOD [10]. Finally, in order to identify *Classes* we use NLIWOD [10]. Below we discuss these systems in more detail.

DBpedia Spotlight is a tool for automatically annotating mentions of DBpedia resources in natural language text [29]. The DBpedia Spotlight first detects possible phrases that are later linked to DBpedia resources. A generative probabilistic model is then applied to disambiguate the detected phrases. Finally, an indexing process is applied to the detected phrases to efficiently extract the corresponding entities from DBpedia [29]. DBpedia Spotlight also allows users to tune the values of important parameters such as the confidence level and support range to get the trade-off between the coverage and accuracy of the detected resources.

TagMe is a tool that on-the-fly identifies meaningful substrings in an unstructured text and links each of them to a pertinent Wikipedia page in an efficient and effective way [30]. TagMe shows good performance especially when annotating texts that are short and poorly composed. This feature of TagMe makes it ideal for question answering tasks. Moreover, TagMe was shown to achieve the best performance on the LC-QuAD dataset among all the available tools used for entity mapping tasks [7].

EARL is a tool for resource and property mapping as a joint task. EARL uses two strategies. The first one is based on reducing the problem to an instance of the Generalized Travelling Salesman problem and the second one uses machine learning in order to exploit the connection density between nodes in the knowledge graph [31]. Both strategies are shown to produce good results for entity and relationship mapping tasks.

Falcon also performs joint resource and property mapping. Falcon shows good performance especially on short texts because it uses a light-weight linguistic approach relying on a background knowledge graph. It uses the context of resources for finding properties and it utilizes an extended knowledge graph created by merging entities and relationships from various knowledge sources [32]. Falcon outperforms other tools and does not require training data, which makes it ideal for a QA system.

RNLIWOD is a tool for mapping properties and classes in the given text. It is shown to have the best overall performance on the LC-QuAD dataset, although its overall macro performance is poor. Therefore, RNLIWOD is augmented with a dictionary of predicates and classes in the question analysis step along with their label information. As a result, the coverage of predicates and classes measured by RNLIWOD increases, which finally leads to an improvement of the overall performance [10].

**Fig. 3:**
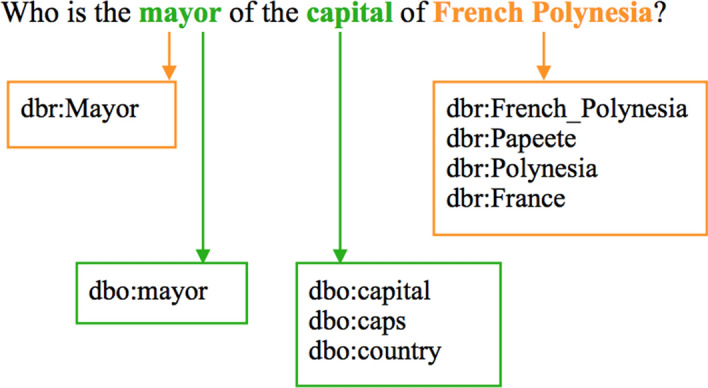
The phrase mapping result for the example question: “Who is the mayor of the capital of French Polynesia?”.* dbo * DBpedia ontology,* dbr* DBpedia resource

Fig. 3 shows the mapped resources, properties and classes for the example question: “Who is the mayor of the capital of French Polynesia?”

### Query generation

As discussed in "Question type classification" section, the component *Question Type Generation* is responsible for determining if a question falls into the category ‘List’, ‘Count’ or ‘Boolean’. This component determines the ‘SELECT’ clause in the SPARQL query. The next step in constructing a SPARQL query is to determine the ‘WHERE’ clause, which is the goal of the component *Query Generation* that we discuss next.

Recall that a SPARQL query is comprised of graph patterns in the form of <subject, predicate, object> triples, where each subject, predicate and object may be a variable. Therefore, the purpose of the query generation step is to construct a set of such triples. These triples are generated based on the output of the mapped resources, properties and classes provided by the component *Phrase Mapping*. Finally, the ‘WHERE’ clause of the SPARQL query is constructed.

In order to find desired RDF triples, all possible combinations of mapped resources, properties and classes are examined [9]. For instance, dbr:French_Polynesia is a mapped *resource* and dbo:capital is a mapped *property* in the example question “Who is the mayor of the capital of French Polynesia?”. The corresponding triple pattern $\texttt {<dbr:French\_Polynesia}\,\texttt {dbo:capital}\,\texttt {?uri>}$ is added to set *S* of all possible triples as it exists in the underlying knowledge graph. Since dbr:France is another mapped resource and dbo:country is another mapped property, the corresponding triple pattern ${\texttt {<?uri}}\,\texttt {dbo:country}\,\texttt {dbr:France>}$ is also added to set *S* of all possible triples as it exists in the underlying knowledge graph.

In more complex SPARQL queries, more than one variable may be involved. Therefore, set *S* is extended by adding the relationship to a new variable [9]. For example, the triple pattern $\texttt {<dbr:French\_Polynesia}\,\texttt {dbo:capital}\,\texttt {?uri>}$ in *S* can be extended by adding another triple pattern $\texttt {<?uri}\,\texttt {dbo:mayor}\,\texttt {?uri'>}$ because dbo:mayor is one mapped property in the example question and such relationship exists in the underlying knowledge graph. The triple pattern $\texttt {<?uri}\,\texttt {dbo:country}\,\texttt {dbr:France>}$ can be extended by adding $\texttt {<?uri'}\,\texttt {dbo:mayor}\,\texttt {?uri>}$ to *S* for the same reason.

We choose to examine only the subgraph containing the mapped resources and properties instead of traversing the whole underlying knowledge graph. As a result, our approach dramatically decreases the computation time compared to [9]. By considering the whole knowledge graph instead, we would have precision and execution time performance drawbacks. For example, one drawback is that the time needed to execute all the possible entity-property combinations increases significantly with the number of properties. As a result, the number of plausible queries to be considered will significantly increase too, and consequently, the time to compute the similarity between questions and SPARQL queries will also increase.

A list of triples needs to be selected from set *S* to build the ‘WHERE’ clause in the SPARQL query. However, the output of the mapped resources, properties and classes from the phrase mapping step may be incorrect and some of them may be unnecessary. Therefore, instead of only choosing the combination of triples which contains all the mapped resources and properties and has the maximum number of triples, combinations of any size are constructed from all triples in *S* as long as such relationship exists in the underlying knowledge graph. For example, ($\texttt {<dbr:French\_Polynesia}\, \texttt {dbo:capital}\,\texttt {?uri>}$ , $\texttt {<?uri}\,\texttt {dbo:mayor}\,\texttt {?uri'>}$) is one possible combination and ($\texttt {<?uri}\,\texttt {dbo:country}\,\texttt {dbr:France>}$, $\texttt {<?uri'}\,\texttt {dbo:mayor}\,\texttt {?uri>}$ ) is another possible combination. Given the question type information, each possible combination can be used to build one SPARQL query. As a result, many possible candidate queries are generated for each input question.

Algorithm 1 summarizes the process of constructing set *S* of all possible triples and set *Q* of all possible queries, where $E'$ is the set of all mapped resources, $P'$ is the set of all mapped properties and *K* is the underlying knowledge graph. The basic idea of generating all possible triple patterns is taken from previous research [9]. However, we improve that approach to be able to generate more possible WHERE clauses and thus, to be able to handle more complex queries (see lines 15–24 of the algorithm below).

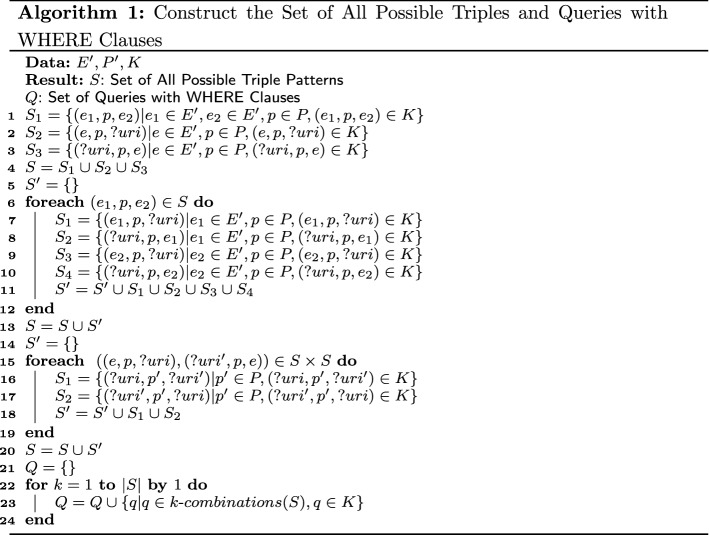



### Query ranking

In the previous step *Query Generation*, we generated a number of candidate queries for each natural language question. The next step is to rank the candidates and to select the most plausible queries. We follow the approach proposed in [9]. It relies on Tree-structured Long-Short Term Memory (Tree-LSTM) [12]. In the following we give a high level account of the method. For technical details we refer the reader to the original publications.

#### Basic idea for ranking

There is an intrinsic tree-like structure in both SPARQL queries and natural language questions. We adopt the basic assumption that the syntactic similarity between between the queries and the input question can be used for ranking. Since the desired query should capture the intention of the input question, the candidate queries that are syntactically most similar to the input question should rank highest.

**Fig. 4:**
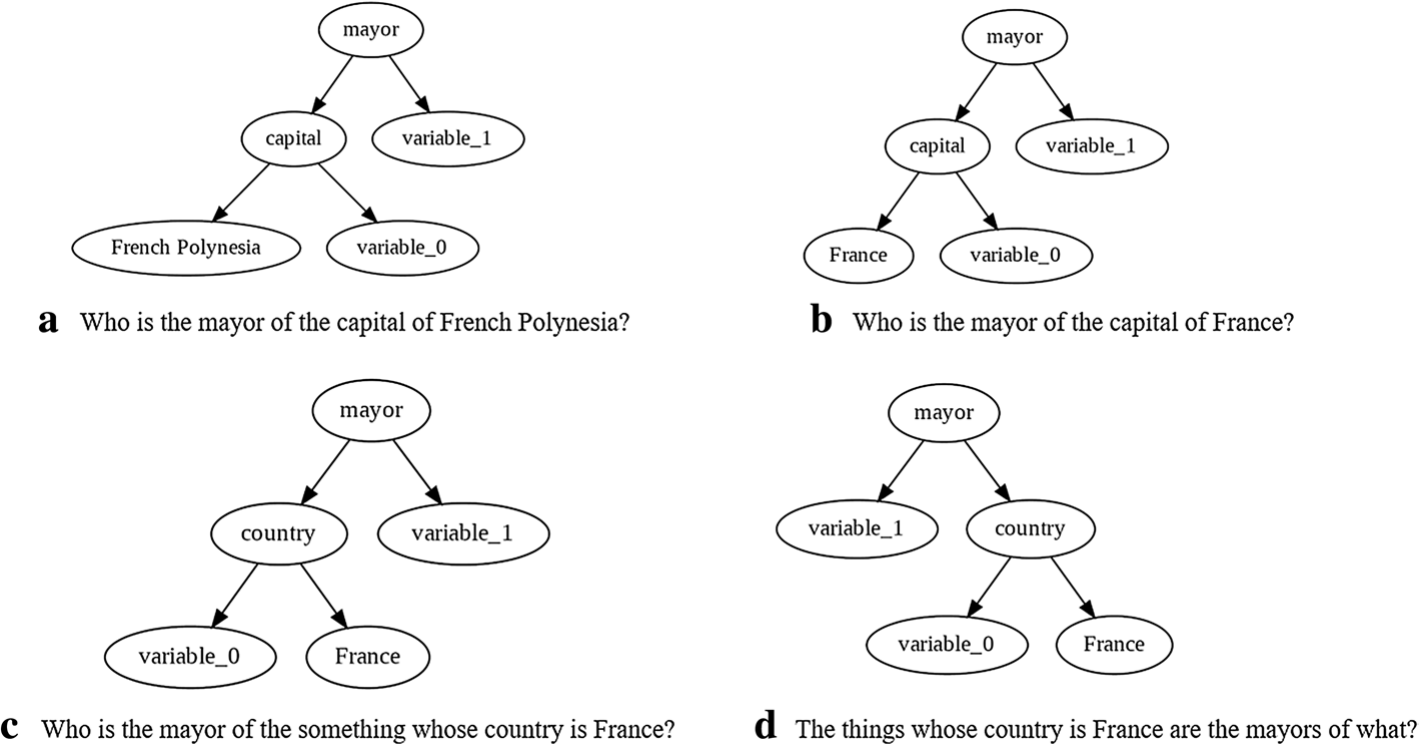
Tree representation of four possible queries of the example question: “Who is the mayor of the capital of French Polynesia?” along with their semantic meaning

As an example, let us revisit the query processing phase with the question: “Who is the mayor of the capital of French Polynesia?”. In the preprocessing phase for the input question, the words corresponding to the mapped resources in the question are substituted with a placeholder. Subsequently, the dependency parse tree of the input question is created (depicted in Fig. 2 for our example). Fig. 4 shows the tree representation of four possible candidate queries for the example question. According to our ranking approach, the first query has the highest similarity among all possible candidate queries.

#### Ranking with tree-LSTM

LSTM augments the vanilla Recurrent Neural Network (RNN) structure with memory cells. Thus, it preserves sequence information over longer time periods. We measure the similarity between candidate queries and the input question based on Tree-LSTM [12]. Standard LSTM operates on a sequential order of the input. Tree-LSTMs take into account the tree representation. More specifically, Tree-LSTM incorporates information not only from an input vector but also from the hidden states of arbitrarily many child units. In contrast, standard LSTM works only with the hidden state of the previous time step. Thus, Tree-LSTM accommodates sentence structure better. Indeed, Tree-LSTM has been shown empirically to outperform strong LSTM baselines in tasks such as predicting semantic relatedness [12].

We use Tree-LSTM to map the input question and the candidate queries to latent space (i.e. numerical vectors), and then compute the similarity between the vectors. More specifically, the dependency parse tree of the natural language question is mapped to latent space via a Tree-LSTM, denoted by *Query Tree-LSTM * in [9]. The tree representations of the candidate queries are mapped to latent space via a different Tree-LSTM denoted *Question Tree-LSTM*. For each sentence pair the similarity score is computed using a neural network that considers both the distance and angle between the vectors in latent space. As a cost function, we use the regularized Kullback–Leibler (KL) divergence between the predicted and the target distributions. Since the goal is to select the candidate query which is most similar to the original natural language question, we pick the sentence pair with the highest similarity. For technical details we refer to the original article [12].

## Results

In this section we describe the experimental evaluation of our system. In order to make our experiments reproducible, we provide our source code for download at https://github.com/Sylvia-Liang/QAsparql. We ran the experiments on two well-established real-world data sets-the Open Challenge on Question Answering over Linked Data Challenge (QALD) [33] and the Large-Scale Complex Question Answering Dataset (LC-QuAD) [34]. Our results show that our QA system outperforms the state-of-art systems by 15% on the QALD-7 dataset and by 48% on the LC-QuAD dataset.

### Evaluation datasets

The LC-QuAD dataset consists of 5000 “question-SPARQL query pairs” that cover 5042 resources and 615 properties [34]. Among the 5000 SPARQL queries in LC-QuAD, only 18% are simple questions, and the remaining questions either involve more than one triple, or involve the COUNT or ASK keyword or both. Moreover, 18.06% questions contain a ‘COUNT’ aggregator, and 9.57% are ‘Boolean’ questions.

The QALD dataset is not one single benchmark but a series of evaluation challenges for Question Answering systems over linked data. The latest version of QALD, which has published the results, is the 7th Question Answering over Linked Data Challenge (QALD-7) [35]. The training dataset of QALD-7 contains 215 questions. Among these 215 questions, 7 questions contain a ‘COUNT’ aggregator, 28 questions are Boolean questions and the remaining 180 questions belong to the type of ‘List’ questions, i.e. they return a list of resources as an answer.

### Evaluation systems

We could only evaluate and compare with QA systems that have either their source code publicly available or tested their approach with the existing benchmark datasets such as LC-QuAD or QALD that we also use in our paper. Finally, by considering the aforementioned, we were able to compare our system with the following state-of-art *SPARQL-based QA systems*: WDAqua-core1 [22], ganswer2 [35], WDAqua [21] and Frankenstein [10]. The reasons for choosing the four SPARQL-based systems for our comparison are as follows. According to the QALD-7 paper [35], the two systems WDAqua [21] and ganswer2 [35] achieved the highest performance on the QALD-7 dataset. According to [22], the system WDAqua-core1 shows the best performance on the LC-QuAD dataset. Finally, we would like to compare with the results from SQG. However, the publication [9] only provides the score of the Query Generation component instead of the performance of the whole end-to-end QA pipeline. Therefore, we instead compare against the state-of-the-art system Frankenstein [10] since it also uses a modular framework that inspired our design.

### Evaluation metrics

In order to compare the performance of our QA system with other published systems, we compared *recall*, *precision* and $F_1$-*Score*, which are calculated for each question *q* as follows:

1 $$\begin{aligned} precision(q)= & {} \frac{number\ of\ correct\ system\ answers\ for\ q}{number\ of\ system\ answers\ for\ q} \end{aligned}$$

2 $$\begin{aligned} recall(q)= & {} \frac{number\ of\ correct\ system\ answers\ for\ q}{number\ of\ benchmark\ answers\ for\ q} \end{aligned}$$

3 $$\begin{aligned} F_1-score= & {} 2 \times \frac{recall(q) \times precision(q)}{recall(q) + precision(q)} \end{aligned}$$

The macro-average precision, recall and $F_1$-score are calculated as the *average* precision, recall and $F_1$-score values for all the questions, respectively.

### Evaluation parameters

In the question type classification component, the LC-QuAD dataset was split into 80% / 20% for the training dataset and test dataset, respectively. The Random Forest Classifier was trained on the training dataset. As parameter values we used 150 estimators, a maximum depth of tree of 150, and the criterion *Gini*.

In the query ranking component, the LC-QuAD dataset was split into 70%/20%/10% for the training dataset, validation dataset, and test dataset, respectively. The parameters of the Tree-LSTM model were tuned based on the validation dataset. The values of hyperparameters used in the query ranking step are summarized in Table 1. The input vector is a 300-dimensional word vector which is embedded using pre-trained FastText embedding models [36]. We used a gradient-based Adagrad Optimizer [37] with a mini batch size of 25 examples. KL divergence was used as the loss function, which provides a useful distance measure for continuous distributions and is often useful when performing direct regression over the space of (discretely sampled) continuous output distributions [38].

**Table 1:** Hyper-parameter values of Tree-LSTM

Parameter	Value
Input dimensions	300 × 1
LSTM memory dimensions	150 × 1
Epochs	15
Mini batch size	25
Learning rate	1 × $10^{-2}$
Weight decay (Regularization)	2.25 × $10^{-3}$
Dropout	0.2
Loss function	Kullback-Leibler divergence loss
Optimizer	Adagrad optimizer
Learning rate scheduler	Stepwise learning rate decay
Step learning rate step size	Once every 2 epochs
Step learning rate decay	0.25

### Performance evaluation

#### Question type classification

In the first part of our experiments we focused on the question type classification. We tested various machine learning methods including Support-Vector Machine (SVM), Random Forest and Tree-LSTM to classify the questions of the two datasets. As shown in Table  2, the Random Forest classifier achieves the highest accuracy on both datasets. Note that the deep learning model Tree-LSTM does not outperform simple classical machine learning models such as SVM and Random Forest for this specific classification task.

**Table 2:** Question type classification performance on LC-QuAD and QALD-7 datasets for various models

Dataset	Accuracy score		
	SVM	Random forest	Tree-LSTM
LC-QuAD	0.986	0.995	0.987
QALD-7	0.937	0.958	0.930

Here we analyze the results for the Random Forest in more detail. In particular, we are interested in the classification accuracy for the three different query types. Let us first start with the LC-QuAD dataset. Table 3 shows the precision, recall and $F_1$-score for each question type. For the LC-QuAD dataset we achieve the highest $F_1$-score for list queries, followed by Boolean and count queries. For the QUALD-7 dataset, the $F_1$-score for list queries is again the highest, while for Boolean queries it is the lowest.

**Table 3:** Question type classification performance on LC-QuAD dataset

Question	LC-QuAD dataset		
Type	Precision	Recall	$F_1$-score
List	0.9945	0.9990	0.9967
Count	0.9944	0.9674	0.9807
Boolean	0.9969	0.9848	0.9908

The question type classification accuracy results on the LC-QuAD dataset are as follows: 99.9% for ‘List’ questions, 97% for ‘Count’ questions, and 98% for ‘Boolean’ questions. These high accuracy values are due to the generation mechanism of the LC-QuAD dataset. This dataset is generated by converting SPARQL queries to Normalized Natural Question Templates (NNQTs) which act as canonical structures. Afterwards, natural language questions are composed by manually correcting the generated NNQTs [34]. Therefore, the questions in LC-QuAD contain much fewer noisy patterns compared to other collected natural language questions. As a result, the performance of the Random Forest Classifier on LC-QuAD dataset is quite satisfactory.

When considering the QALD-7 dataset for the question type classification, our approach performed slightly worse than with the LC-QuAD dataset as shown in Table 4. The accuracy for ‘List’ questions is 97%, for ‘Count’ questions 93% and for ‘Boolean’ questions 86%. The reduction in performance is mainly due to the different qualities of the datasets. For instance, the QALD-7 dataset contains questions with richer characteristics such as ‘Boolean’ questions starting with ‘Are’ or ’Was’. However, the LC-QuAD dataset contains very few such ‘Boolean’ questions, which results in the dramatic decrease in the accuracy for ‘Boolean’ questions.

**Table 4:** Question type classification performance on QALD-7 dataset

Question	QALD-7 dataset		
Type	Precision	Recall	$F_1$-score
List	0.9830	0.9665	0.9746
Count	1.0000	0.9310	0.9643
Boolean	0.5000	0.8571	0.6316

#### End-to-end system evaluation

In this part of experiments, we analyze the overall performance of our proposed end-to-end system. Our system receives natural language questions as input, translates them into corresponding SPARQL queries and returns answers extracted from the underlying knowledge graph. The following reported performance values are calculated based on the returned answers.

**Fig. 5:**
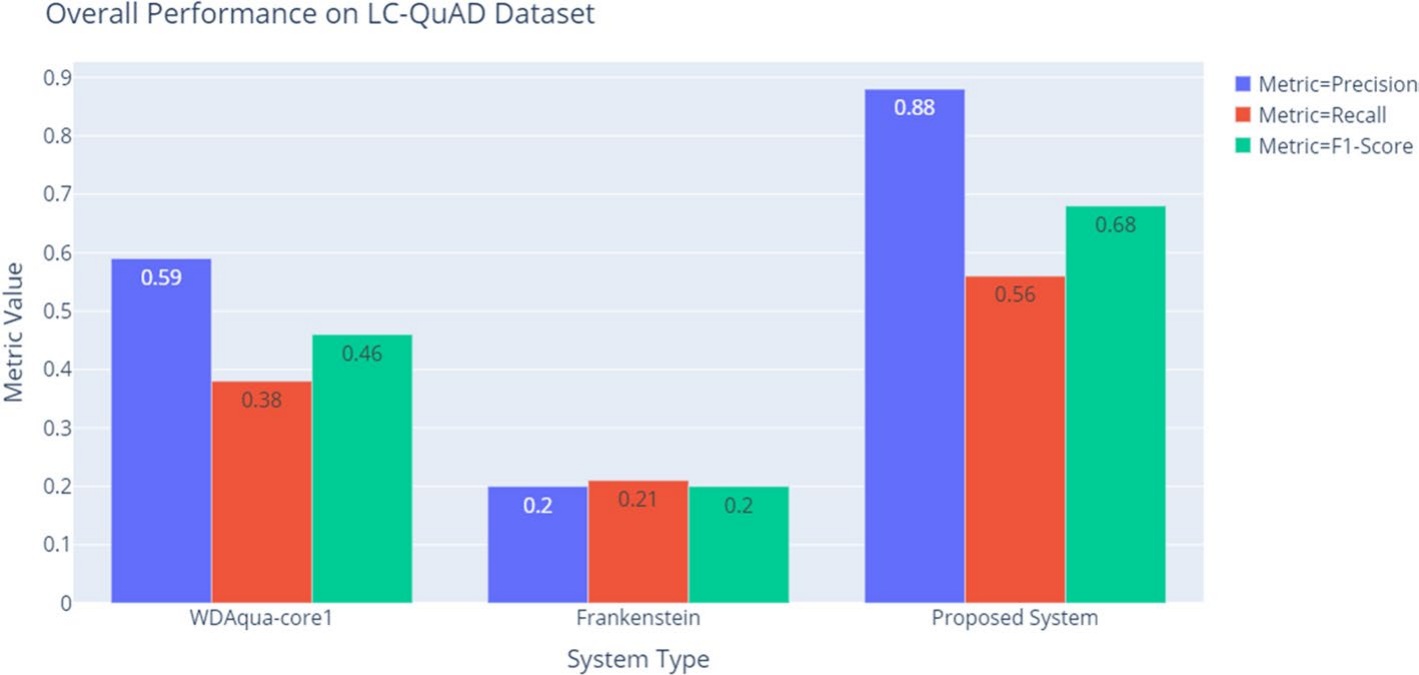
End-to-end performance on the LC-QuAD dataset

Table 5 and Fig. 5 show the comparison of the performance of our QA system with published result of the state-of-art systems WDAqua-core1 [22] and Frankenstein [10]. This comparison result demonstrates that our proposed QA system significantly outperforms the state-of-art QA systems on the LC-QuAD dataset. We tested our QA system on 2430 questions in the LC-QuAD dataset which are still applicable to the latest SPARQL endpoint version (2019-06).

**Table 5:** End-to-end performance on the LC-QuAD dataset

Evaluation	Models		
	WDAqua-core1	Frankenstein	Proposed system
Precision	0.59	0.20	0.88
Recall	0.38	0.21	0.56
$F_1$-score	0.46	0.20	0.68

**Fig. 6:**
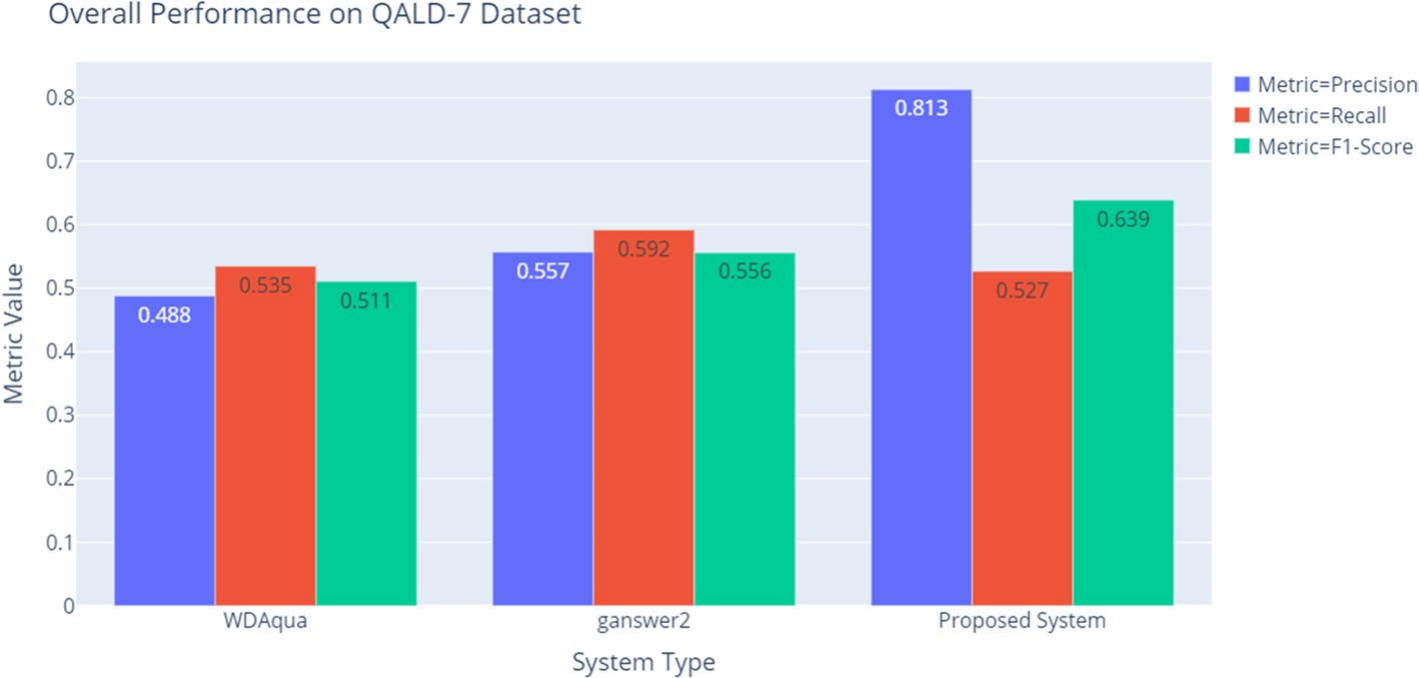
End-to-end performance on the QALD-7 dataset

Table 6 and Fig. 6 show that on the QALD-7 dataset our QA system also significantly outperforms the state-of-art systems WDAqua [21] and ganswer2 [35].

**Table 6:** End-to-end performance on the QALD-7 dataset

Evaluation	Models		
	WDAqua	ganswer2	Proposed system
Precision	0.488	0.557	0.813
Recall	0.535	0.592	0.527
$F_1$-score	0.511	0.556	0.639

Our in-depth analysis of the failed questions shows that no SPARQL query was generated for 968 questions in LC-QuAD datset and 80 questions in QALD-7 dataset. Most of these failures were related to the phrase mapping step where the required resources, properties or classes could not be detected.

For instance, most of these failures are related to detecting properties implicitly stated in the input question. In such cases, the properties required to build the SPARQL query cannot be inferred from the input question. For example, consider the question “How many golf players are there in Arizona State Sun Devils?”. The correct SPARQL query should be:

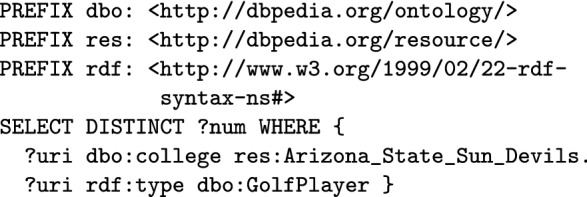



The property http://dbpedia.org/ontology/college is necessary to build the correct SPARQL query but it is impossible to detect it solely from the input question. Therefore, the bottleneck of designing QA systems over knowledge graphs lies in the phrase mapping step, i.e detecting the corresponding resources, properties and classes in the underlying knowledge graph.

**Fig. 7:**
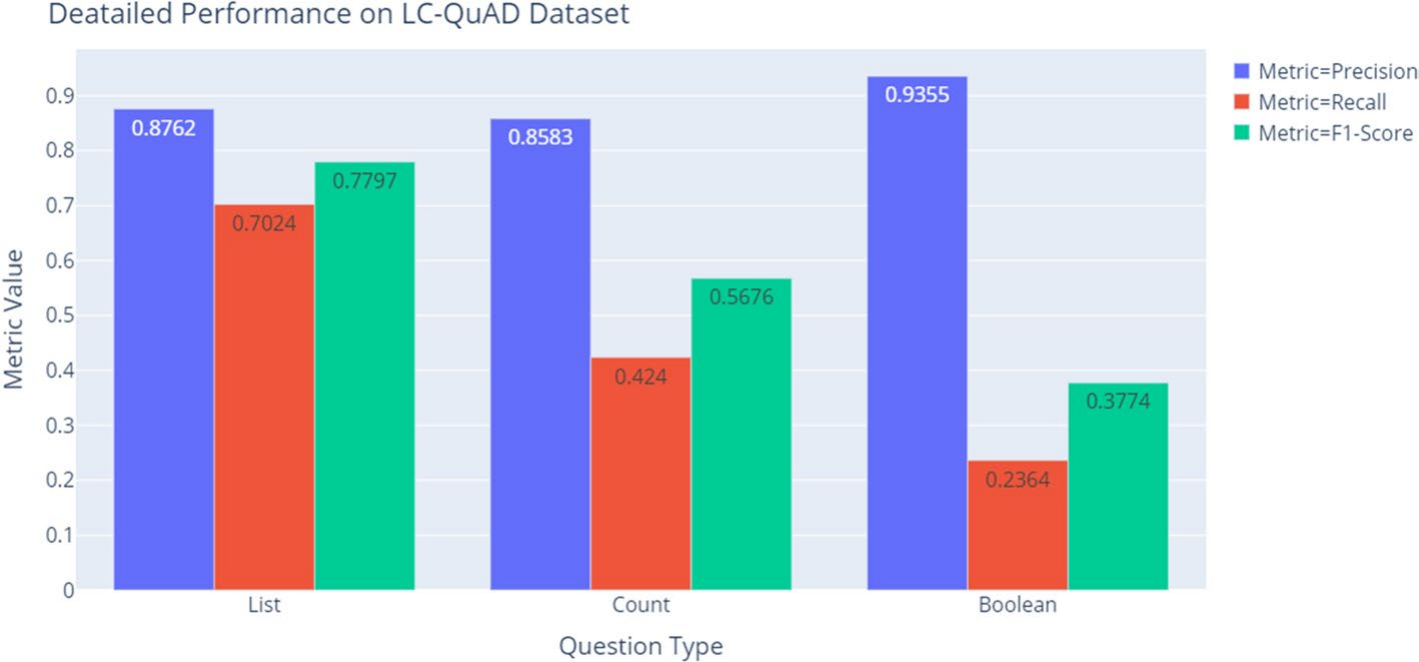
Performance of each question type on LC-QuAD dataset

**Fig. 8:**
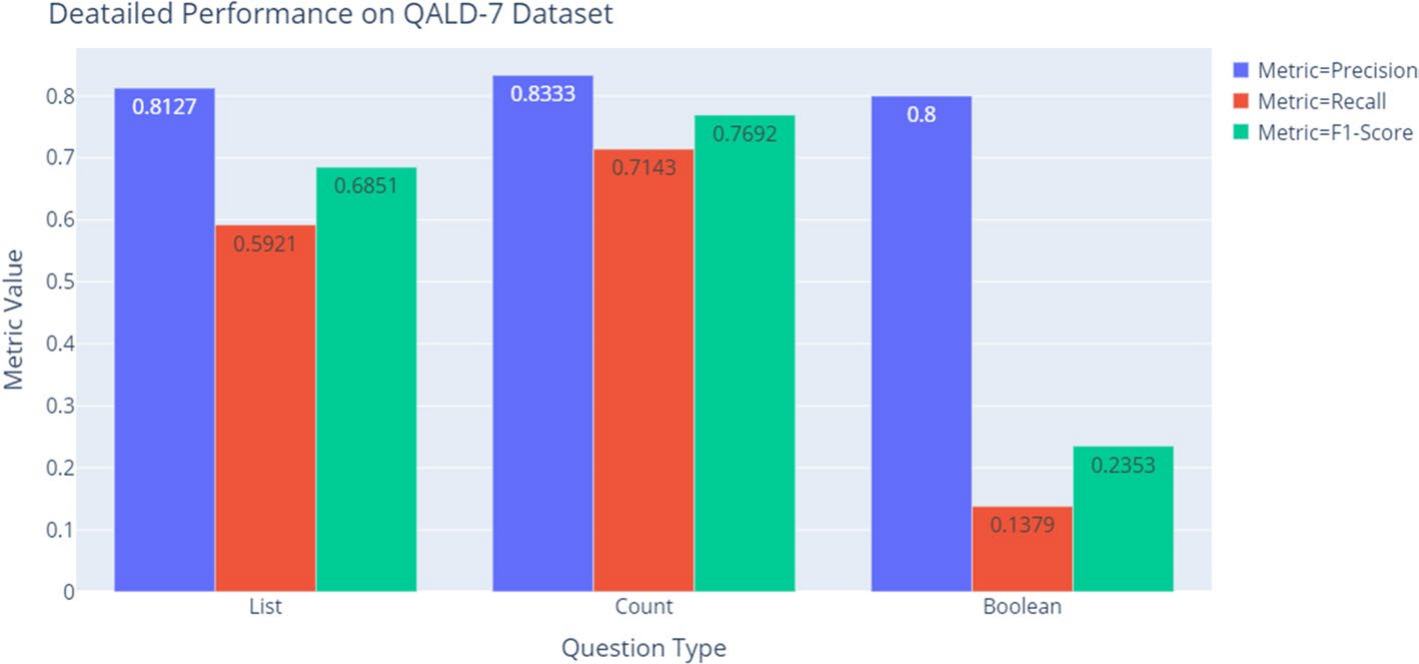
Performance of each question type on QALD-7 dataset

The previous experiments showed the end-to-end performance of our system. We will now show more detailed performance analysis based on the question type of the natural language questions, which are presented in Tables  7, 8, Figs. 7, 8. Both Tables  7, 8 shows that the performance on ‘List’ questions is much better than the performance on ‘Boolean’ questions. Low recall for ‘Boolean’ questions might be caused by the intrinsic structure of the SPARQL query. For instance, the question “Is Tom Cruise starring in Rain Man?” has the following SPARQL query:

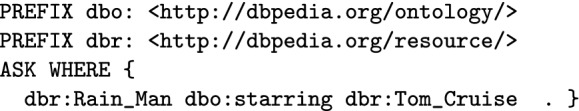



According to the input question, the generated query should be ${\texttt {<dbr:Tom\_Cruisedbo:starringdbr:Rain\_Man>}}$. However, the correct triple pattern is the opposite, i.e. ${\texttt {<dbr:Rain\_Man dbo:starring dbr:Tom\_Cruise>}}$. It is difficult to distinguish between these two triples solely based on the current small training dataset. Therefore, more training data of ‘Boolean’ questions are needed to fully capture the characteristics of such questions and queries. In addition, advanced query ranking mechanisms which could better capture the intention behind questions could also be useful in improving the recall on ‘Boolean’ questions.

The high $F_1$-score of ‘Count’ questions in the QALD-7 dataset does not have much affect because there are only 7 questions of the ‘Count’ type in the QALD-7 dataset. The reason for the low recall of ‘Count’ questions in LC-QuAD dataset might be the high complexity of the SPARQL queries. Most queries with the ’COUNT’ keyword are quite complex because they tend to contain more than one triple and variable in the WHERE clause. However, the number of ‘Count’ questions in the training dataset is relatively small as there are only 658 ‘Count’ questions in LC-QuAD dataset. Therefore, more training data is required in order to fully learn the characteristics of those complex queries.

**Table 7:** Performance of each question type on LC-QuAD dataset

Question	LC-QuAD dataset		
Type	Precision	Recall	$F_1$-score
List	0.8762	0.7024	0.7797
Count	0.8583	0.4240	0.5676
Boolean	0.9355	0.2364	0.3774

**Table 8:** Performance of each question type on QALD-7 dataset

Question	QALD-7 dataset		
Type	Precision	Recall	$F_1$-score
List	0.8127	0.5921	0.6851
Count	0.8333	0.7143	0.7692
Boolean	0.8000	0.1379	0.2353

## Discussion

### Conclusions

This paper presents a novel approach to constructing QA systems over knowledge graphs. Our proposed QA system first identifies the type of each question by training a Random Forest model. Then, an ensemble approach comprised of various entity recognition and property mapping tools is used in the phrase mapping task. All possible triple patterns are then extracted based on the mapped resources, properties and classes. Possible SPARQL queries are constructed by combining these triple patterns in the query generation step. In order to select the correct SPARQL query among a number of candidate queries for each question, a ranking model based on Tree-LSTM is used in the query ranking step. The ranking model takes into account both the syntactical structure of the question and the tree representation of the candidate queries to select the most plausible SPARQL query which represents the correct intention behind the question. Experimental results demonstrate that our proposed QA system outperforms the state-of-art result by 15% on the QALD-7 dataset and 48% on the LC-QuAD dataset, respectively.

The advantage of our QA system is that it requires neither any laborious feature engineering, nor does it require a list of heuristic rules mapping a natural language question to a query template and then to a SPARQL query. In this sense, our system could avoid the over-fitting problem, which usually arises when defining the heuristic rules for converting from natural language to a SPARQL query. In addition, our proposed system can be used on large open-domain knowledge graphs and handle noisy inputs, as it uses an ensemble method in the phrase mapping task, which leads to a significant performance improvement. What is more, each component in our QA system is reusable and can be integrated with other components to construct a new QA system that further improves the performance. This proposed system can be easily applied to newly unseen domains because the question type classification model and the query generation model do not require any domain specific knowledge.

One important design question might concern our choice of a modular architecture, rather than an end-to-end system. The reason behind this choice is that the modular approach makes the QA system more independent and less susceptible to data schema changes. An end-to-end system often needs to be re-trained due to potentially frequent changes of the underlying database. However, in a modular system, only one or two components will be affected by the changes in the underlying database, and as a result, the training time and computing effort for updating the modular system is much smaller than an end-to-end system. In addition, in order to match the changed underlying database, the adjustment of the architecture used by a modular system will also be much smaller compared to the end-to-end system.

Nowadays, many graph databases such as DBpedia and UniProt provide a SPARQL endpoint for end users to access information. However, end users have to master the SPARQL query language and the structure of the database in order to utilize the provided SPARQL endpoint. With the help of the proposed system, which can automatically translate natural language questions into corresponding SPARQL queries, non-tech savvy users can now take advantage of the large and complex graph databases much more efficiently and easily.

### Future work

Currently available training datasets contain only three types of questions and therefore the diversity of training data is limited. In reality, many more types of questions are commonly used. Among the commonly used SPARQL operators, which were not considered here, are FILTER, LIMIT, ORDER, MIN, MAX, UNION, etc. Collecting complex questions containing the listed operators to improve both the size and the quality of the training dataset is one obvious direction for this work. The number of questions for each type should be relatively homogeneous in the training dataset. Moreover, multiple expressions for the same question should also be developed to increase the size and variety of the training dataset and to improve the system performance.

Another possible future research direction is to convert the current QA system into an architecture similar to distributed systems. Efforts could be made to integrate multiple knowledge graphs in order to return the correct answers. For instance, one complex question may require information from multiple resources such as both DBpedia and Freebase [39]. Hence, the current system could be extended to multiple knowledge graphs by detecting the related knowledge graph, building sub-queries for each possible knowledge graph and finally returning the correct answer by composing the complete query from the generated sub-queries.

As mentioned earlier, a major strength of the proposed QA system is the modular framework. Consequently, the performance of the whole system could be increased by improving each component model. Future efforts could be made by either upgrading the current component models or replacing current models by more advanced ones. For instance, when more types of questions are available in the training dataset, the question type classification component might be replaced by more complex machine learning models in order to achieve higher classification accuracy.

Our current ensemble method for phrase mapping returns the union of all the individual methods, thereby potentially increasing the true positive rate at the phrase mapping and query generation steps. The increase may come at the cost of more false positive candidate queries. However, these should be filtered out at the query ranking step. A conservative ensemble method can be obtained by some consensus criterion over the individual phrase mappers. As future work, a parameter could be introduced to move between union and consensus to control for the precision-recall trade-off.

Among the five components in the system, currently only the phrase mapping component depends on the underlying knowledge graph. Specifically speaking, the phrase mapping model used in this paper performs well on DBpedia but not on other knowledge graphs because it uses many pre-trained tools for DBpedia. In order to make this system fully independent of the underlying knowledge graph, and for it to be easily transferable to a new domain, the models used in this component could be changed to more general models. For instance, DeepType [40] could map resources in Wikidata [41], Freebase and YAGO2 [42]. If no pre-trained phrase mapping models are available for a specific knowledge graph, one simple model is to measure the similarity between the phrases in question and the labels of resources in the knowledge graph. In order to improve the accuracy of this simple approach, specific tailoring for each knowledge graph would be required.

## Data Availability

Not applicable.

## Footnotes

1 https://www.w3.org/TR/2014/REC-rdf11-concepts-20140225/#dfn-iri.

2 https://www.w3.org/TR/2014/REC-rdf11-concepts-20140225/#dfn-literal.
